# α/β-Hydrolase Domain-Containing 6 (ABHD6)— A Multifunctional Lipid Hydrolase

**DOI:** 10.3390/metabo12080761

**Published:** 2022-08-18

**Authors:** Lisa-Maria Pusch, Lina Riegler-Berket, Monika Oberer, Robert Zimmermann, Ulrike Taschler

**Affiliations:** 1 Institute of Molecular Biosciences, NAWI Graz, University of Graz, 8010 Graz, Austria; 2 BioTechMed-Graz, 8010 Graz, Austria; 3 Field of Excellence BioHealth, University of Graz, 8010 Graz, Austria

**Keywords:** ABHD6, lipid hydrolase, monoacylglycerol, bis(monoacylglycerol)phosphate, inflammation, metabolic syndrome, insulin signaling

## Abstract

α/β-hydrolase domain-containing 6 (ABHD6) belongs to the α/β-hydrolase fold superfamily and was originally discovered in a functional proteomic approach designed to discover monoacylglycerol (MAG) hydrolases in the mouse brain degrading the endocannabinoid 2-arachidonoylglycerol. Subsequent studies confirmed that ABHD6 acts as an MAG hydrolase regulating cannabinoid receptor-dependent and -independent signaling processes. The enzyme was identified as a negative modulator of insulin secretion and regulator of energy metabolism affecting the pathogenesis of obesity and metabolic syndrome. It has been implicated in the metabolism of the lysosomal co-factor bis(monoacylglycerol)phosphate and in the surface delivery of α-amino-3-hydroxy-5-methyl-4-isoxazolepropionic acid-type glutamate receptors. Finally, ABHD6 was shown to affect cancer cell lipid metabolism and tumor malignancy. Here, we provide new insights into the experimentally derived crystal structure of ABHD6 and its possible orientation in biological membranes, and discuss ABHD6′s functions in health and disease.

## 1. Introduction

Lipids are components of biomembranes, energy storage molecules, and signaling molecules regulating a variety of biological processes. Considering these essential functions, lipid metabolism must be tightly regulated to achieve homeostasis under changing environmental conditions. The dysregulation of lipid metabolism has been associated with rare monogenetic diseases as well as common disorders including obesity-related diseases and cancer. Therefore, the precise clarification of the molecular pathways regulating lipid metabolism can improve our understanding of lipid-associated pathologies and unveil novel treatment strategies. Lipid synthesis and degradation are catalyzed by multiple enzymes encoded by the mammalian genome. In the postgenomic era, many new lipid hydrolases and acyltransferases have been discovered. However, currently, it is still unclear how many lipid-modifying enzymes exist, and many of the known enzymes remain insufficiently characterized.

Here, we provide a review on the serine hydrolase α/β-hydrolase domain-containing 6 (ABHD6) belonging to the large α/β-hydrolase fold superfamily, first classified in 1992. This protein superfamily is found in all domains of life and comprises lipases, proteases, dehalogenases, esterases, peroxidases, and epoxide hydrolases [1,2]. The structural characteristic of α/β-hydrolases is the core fold consisting of eight mostly parallel strands connected by helices and loops [2,3,4,5]. The loops are carrying the residues of the catalytic triad formed by a nucleophile-like serine within the consensus sequence Sm-X-Nu-X-Sm (Sm = small residue, often a glycine, with alanine or serine also possible, X = any residue, Nu = nucleophile), an acidic residue (aspartate or glutamate), and a histidine [2,3]. Typically, two residues form the oxyanion hole, which is crucial to stabilize the negatively charged transition state during hydrolysis [1,2,4].

ABHD6 was first detected in an activity-based proteomic approach using the general serine hydrolase inhibitor methyl arachidonoyl fluorophosphonate (MAFP), which broadly targets serine hydrolases [6]. Subsequent investigations revealed that it is a typical member of the α/β-hydrolase fold superfamily, and enzyme assays suggested that the enzyme possesses monoacylglycerol (MAG) hydrolase and other activities. ABHD6 is ubiquitously expressed, with its highest expression observed in brain, brown adipose tissue, and the intestine [7,8]. In the last years, substantial progress has been made in the understanding of the role of ABHD6 in health and disease.

## 2. Structure of ABHD6

In 2021, A. Nawrotek, A. Talagas, L. Vuillard, and L. Miallau deposited the experimental structure of human ABHD6 obtained by X-ray crystallography [9]. The structure and experimental details have currently not been described in a publication. As deduced from the deposited coordinate file, ABHD6 harbors a canonical α/β-hydrolase fold in the core as predicted, and presented in previously published homology models and the newest AlphaFold (AF) model [10,11,12]. The deposited experimental protein data bank (PDB) structure spans from residue Arg43 to Leu336, in agreement with a predicted N-terminal transmembrane region omitted in the cloning. This human ABHD6 variant was expressed in *E. coli* and crystals diffracting to 1.8 Å were obtained.

**Figure 1:**
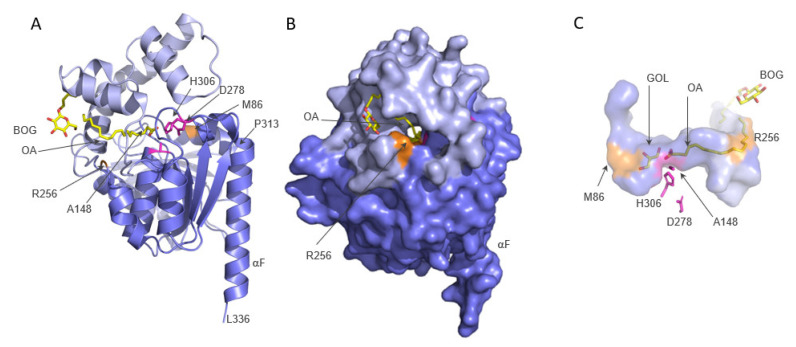
The experimental structure of ABHD6. (**A**) Cartoon representation of ABHD6 complexed with octyl beta-D-glucopyranoside (BOG) and oleic acid (OA), both depicted in yellow. The cap/lid region is colored light blue and the core is slate-blue. The active site residues (please note the S148A exchange) are depicted as magenta sticks. (**B**) Surface representation of (**A**) after approximately 90° rotation to the right with αF facing the back. A tunnel is visible that spans through the entire molecule, approximately between residues M86 and R256 (orange). (**C**) Surface representation of the tunnel between residues M86 and R256 (orange). Octyl beta-D-glucopyranoside (BOG) and oleic acid (OA) are present in chain A; a glycerol molecule (GOL) was fitted into the tunnel only in chain B by the deposition authors. Other residues are omitted for clarity.

The 3D structure (Figure 1) shows a central mostly parallel β-sheet containing eight strands (β1–β8), whereby only the second strand is antiparallel. Two α-helices on one side (αA, αF) and four α-helices (αB, αC, αD, αE) on the other side of the sheet connect the individual strands. A long insertion (Gly175–Ser255) after β6 connects to αD. This region harbors four helices with interspersed loops, and forms a lid that covers the active site as a continuous surface (light blue in Figure 1).

The catalytically active residues in human ABHD6 are Ser148, Asp278, and His306. The experimental structure (PDB code 7OTS) harbors an amino acid exchange Ser148Ala, which renders the hydrolase inactive. Similarly, the amino acid exchange Ser148Tyr in the active serine of human ABHD6 leads to a complete loss of hydrolytic activity [13]. Ala148 is located in the so-called nucleophilic elbow connecting β5 with αC in a sharp turn. Asp278 is in a loop region connecting β7 with αE, and the conserved His306 of the triad is positioned at the loop connecting β8 with αF (Figure 1).

Sequence and structure alignments to human monoglyceride lipase (also monoacylglycerol lipase, MGL) suggest that the peptidic N-Hs of residues Phe80 and Met149 form the oxyanion hole. These main chain N–Hs bind the carbonyl oxygen of the substrate and stabilize the oxyanion of the tetrahedral intermediates as well as the acyl-enzyme during the enzymatic reaction [14].

The experimental structure of ABHD6 contains two molecules (chain A, B) in the asymmetric unit. Chain A was determined in complex with oleic acid (OA) and octyl beta-D-glucopyranoside (BOG), whereas one OA and three glycerol (GOL) molecules were fitted into chain B. Interestingly, a tunnel is visible that spans roughly 23 Å through the entire molecule (Figure 1B,C). Residues of the α/β-hydrolase core line the bottom, and residues of the cap form the roof of the tunnel, respectively. The tunnel of human ABHD6 is rather hydrophobic in proximity to the active site towards the smaller entrance lined by Arg256. Aliphatic residues in this region provide a very good physico-chemical environment for the alkyl chain of the substrate. A rather polar side-pocket branches off from the hydrophobic tunnel in the area where the alkyl chain of OA bends upwards toward the tunnel entrance and reaches the surface. Similar to published MGL structures, the other side of the active site cavity harbors more polar residues. The deposition authors of human ABHD6 could model a molecule of glycerol in addition to OA into the long tunnel of chain B (Figure 1C). With lack of a publication along with the deposited structure, the source of the glycerol molecule is unknown. Glycerol is a common buffer ingredient added during protein purification and used as cryoprotectant in the crystal freezing process, which could explain the glycerol in the structure. Equivalent residues in MGL structures from different organisms have been discussed to interact with the polar glycerol moiety or isopropanol found within this area [15,16,17].

**Figure 2:**
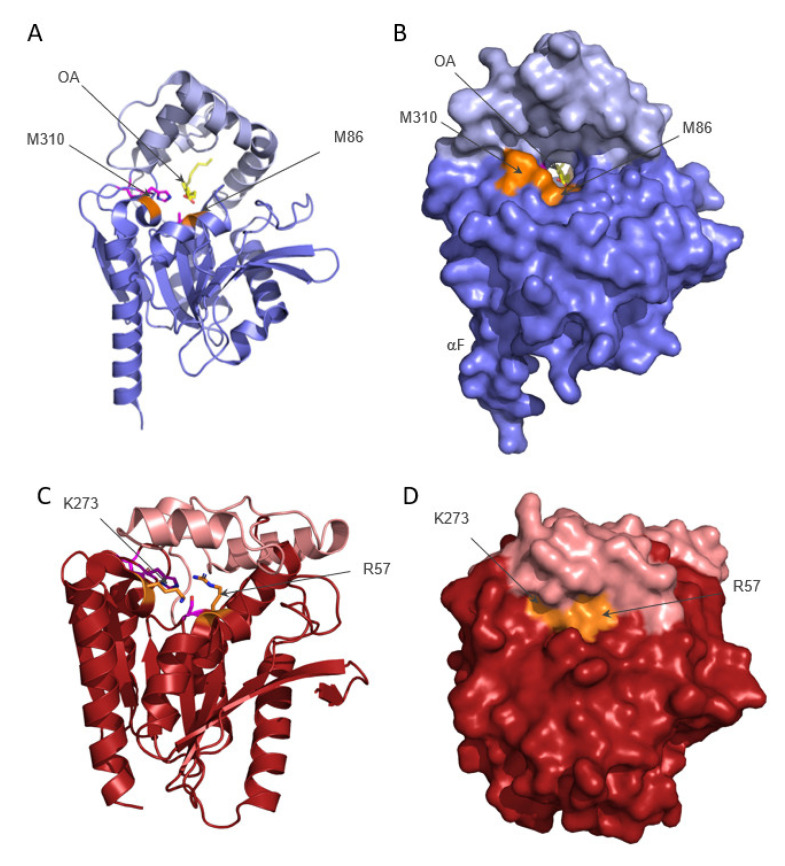
Comparison of the experimental structures of ABHD6 and MGL. (**A**) ABHD6 is complexed with oleic acid (OA, depicted in yellow). The cap/lid region is colored light blue and the core is slate-blue. The active site residues (please note the S148A exchange) are depicted as magenta sticks. (**B**) Surface representation of (**A**). The tunnel is visible and spans through the entire molecule, the rim residues M86 and M310 (orange) are on one face of the molecule, and R256 is located on the other side. (**A**,**B**) These images correspond to an approximately 180° rotation to the left of Figure 1B with αF on the left. (**C**,**D**) Cartoon (**C**) and surface (**D**) representation of human MGL (P20-A297, PDB code 3HJU). Human MGL was aligned to human ABHD6 and presented in the same orientation as ABHD6 in panels 2A and 2B. Residues R57 and K273 are at equivalent spatial positions to K86 and M310 in ABHD6 and are indicated in orange. The lid of human MGL is shown in salmon and the core in dark red. Catalytic residues S122, D239, and H269 are colored magenta.

The tunnel has its opening between the cap and the core region, approximately between residues Arg256 (located after the last helix of the cap) and residues Met86 and Met310 (Figure 1B,C and Figure 2A,B). Met310 is part of the rim of the widely open tunnel entrance. In the structure, Met310 adopts two alternate conformations before Pro313 induces the bend leading to αF. The large opening of the tunnel on the face of Met86/Met310 is mediated in part by (i) the upward bend of the lid in the region Val220 to Ile237 (Figure 2A,B), and in part because (ii) the sidechain of Met86 is pointing inwards, forming the floor of the tunnel. In human MGL (PDB code 3HJU [15]), the corresponding lid region ranges from Arg186 to Arg202 and is positioned much closer by the α/β hydrolase core, thereby closing off the internal cavity of human MGL (Figure 2). Arg57 of human MGL corresponds to the Met86 in human ABHD6 and its sidechain points upwards towards the lid helix, thereby closing off the internal cavity of human MGL. Lys273 of human MGL (corresponding to the rim residue Met310 in human ABHD6) is located before a sharp bend in the helix mediated by Pro276 leading to αF (Figure 2C,D).

**Figure 3:**
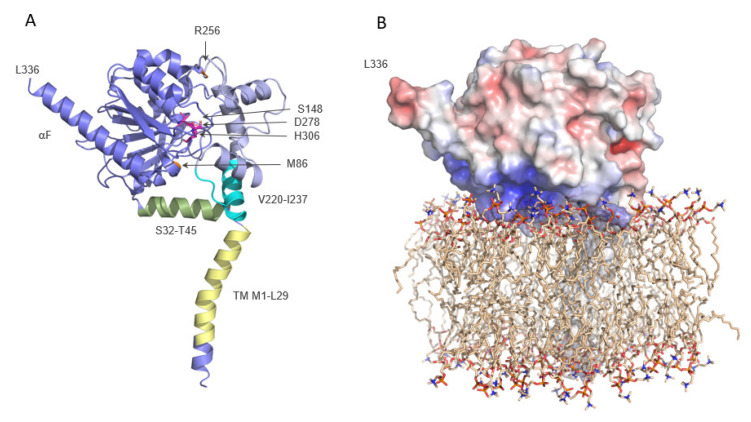
Predicted models of ABHD6 and possible membrane insertion. (**A**) Cartoon representation of ABHD6 AlphaFold model. The cap/lid region is colored light blue and the core is slate-blue. The active site residues are depicted as magenta sticks. Both sides of the tunnel entrance are marked with the residues M86 and R256 in orange. The additional helix (S32-T45) and the cap region (V220-I237) forming a part of the tunnel entrance are depicted in green and cyan, respectively. The transmembrane (TM) helix in light yellow. (**B**) Model of ABHD6 embedded in a di-oleoylphosphatidylcholine (DOPC) membrane. ABHD6 (same orientation as in **A**) is represented in surface mode. Electrostatic surface calculation was performed with the ABPS electrostatics plugin in PyMOL [18]: red—negative and blue—positive. The DOPC membrane model [19,20] is presented as sticks.

In the AF model of human ABHD6, the first 43 amino acids, which are missing in the experimental structure, are also computed (Figure 3A). In the rest of the model, the C-alpha backbone alignment between the AF model and the experimental structure shows a high similarity with a root-mean-square deviation (RMSD) of 0.424Å over 218 aligned residues. Owing to these additional modeled parts, the large entrance to the tunnel is more restricted at the face of Met86 by an N-terminal helix Ser32 to Thr45. Here, the tunnel entrance is reformed by lid region Val220 to Ile237 (as observed in the experimental structure) and this additional helix. Furthermore, the AF model also includes the N-terminal transmembrane (TM) helix that is predicted to stretch out of the molecule in an almost perpendicular orientation to the tunnel entrance. However, the orientation of the TM helix with respect to the rest of the protein has to be interpreted with caution, as reflected in the high predicted aligned error for this region.

Based on the 3D structure and the predicted TM helix, we also generated a first model of ABHD6 embedded in a phospholipid-containing membrane (Figure 3B). In this model, the wide entrance to the tunnel is oriented towards the membrane. The electrostatic surface properties of ABHD6 reveal a rather positively charged face of the protein interacting with the surface of the membrane. This model suggests that human ABHD6 can attach to charged membrane surfaces, and may explain the preference for the negatively charged lipid substrate bis(monoacylglycerol)phosphate (BMP). It should be kept in mind, however, that this model must be interpreted with caution because the relative position of the TM helix to the rest of the protein is uncertain and awaits experimental verification on different membranes.

**Figure 4:**
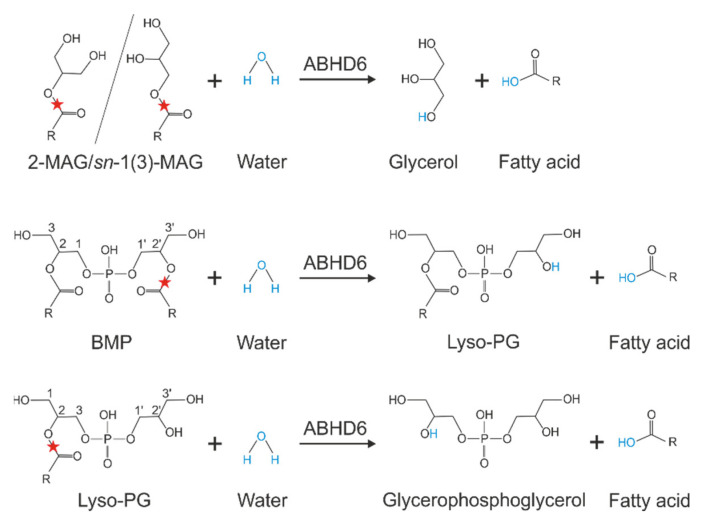
Substrate specificity of ABHD6: ABHD6 is capable of hydrolyzing sn-2 and sn-1(3) monoacylglycerols (MAGs) releasing glycerol and a fatty acid, the phospholipid bis(monoacylglycerol)phosphate (BMP) generating lyso-phosphatidylglycerol (LPG) and a fatty acid, and LPG releasing a fatty acid and glycerophosphoglycerol. The red star indicates the cleavage site and R indicates an acyl chain.

The comparison of ABHD6 with structures from human MGL or from Bacillus species H257 (bMGL; [16]) opens further interesting questions. In some structures, the substrate binding pocket on one side leads to the smaller opening on the other side of the molecule, which was termed the ‘glycerol exit hole’. It has to be noted here that this opening correlates to the wide tunnel entrance (on the face of Met86/Met310) in ABHD6. It was postulated that the smaller reaction product glycerol would leave via this exit path after the first part of the hydrolytic reaction is completed. Clearly, more structural studies are required to determine whether the large tunnel observed in human ABHD6 is a requirement to accommodate the rather large substrate BMP or lyso-phospholipids (compare with Figure 4). This would provide a very distinctive element when comparing MGLs with ABHD6—and possibly other members of the ABHD family, e.g., ABHD12. More studies are needed to experimentally observe the orientation of different uncleaved substrates of ABHD6 in the binding tunnel. It will also be interesting to see whether the large openings of the tunnel on either side can be entirely closed, or whether the lid itself can adopt an open conformation, as observed in some structures of human MGL.

Detailed research is necessary to gain knowledge about the substrate entrance, product exit, and about potential reorientation or conformational changes in the cap during the process of substrate binding and product release.

## 3. Substrate Specificity of ABHD6

ABHD6 acts as a lipid hydrolase and degrades a variety of MAGs esterified with saturated, monounsaturated, and polyunsaturated fatty acids. The highest activities were detected in MAGs esterified with saturated fatty acids (C8:0–C14:0) and arachidonic acid (AA; C20:4). Thereby, ABHD6 showed a preference for the sn-1(3)- over sn-2-isomers [21]. This positional preference was confirmed in a very recent study [22]. In addition to MAGs, ABHD6 cleaves lyso-phospholipids with a preference for lyso-phosphatidylglycerol (LPG), while fully acylated phospholipids are not hydrolyzed [8,13]. Additionally, ABHD6 hydrolyzes BMP at a similar rate as observed for MAGs [13]. This negatively charged phospholipid, also known as lyso-bisphosphatidic acid, is a structural isomer of phosphatidylglycerol and exhibits an unusual sn-1-glycerophosphate-sn-1′-glycerol backbone stereo-configuration, while acylated glycerol backbones of all other mammalian phospholipids exhibit sn-3 configuration (Figure 4). ABHD6 is capable of hydrolyzing BMP independent of its stereo-configuration and does not show positional preferences for fatty acids [13]. Finally, recent observations suggest that ABHD6 is involved in diacylglycerol (DAG) degradation [23].

## 4. Tools for the Investigation of ABHD6 Function

The availability of chemical and genetic tools strongly facilitated the investigation of ABHD6 function in vitro and in vivo. Genetic tools include mutant mouse and rat lines, whereby mouse lines allow the characterization of animals with global and tissue-specific deletion of ABHD6 [24,25,26,27,28,29]. Table 1 summarizes the different animal models and the most important findings of respective studies (Table 1). Furthermore, several small molecule inhibitors for ABHD6 have been developed, which can be used to investigate ABHD6 function in cell culture and in in vivo studies (reviewed in [30]). In 2017, Abide Therapeutics patented compounds that inhibit ABHD6 and lipoprotein-associated phospholipase A2 (patent WO2017059135A1) for the treatment of various diseases. All reported inhibitors interact irreversibly with the enzyme forming adducts with the nucleophilic Ser148 of ABHD6. These inhibitors are active in the nanomolar range and show selectivity over other serine hydrolases. Notably, a very recent study reported the development of potent ABHD6 inhibitors with more than 1000-fold selectivity over other endocannabinoid (EC)-degrading enzymes [31]. Finally, ABHD6 can be efficiently silenced using antisense oligonucleotides (ASO) [8].

## 5. The Role of ABHD6 in Endocannabinoid Signaling

**Figure 5:**
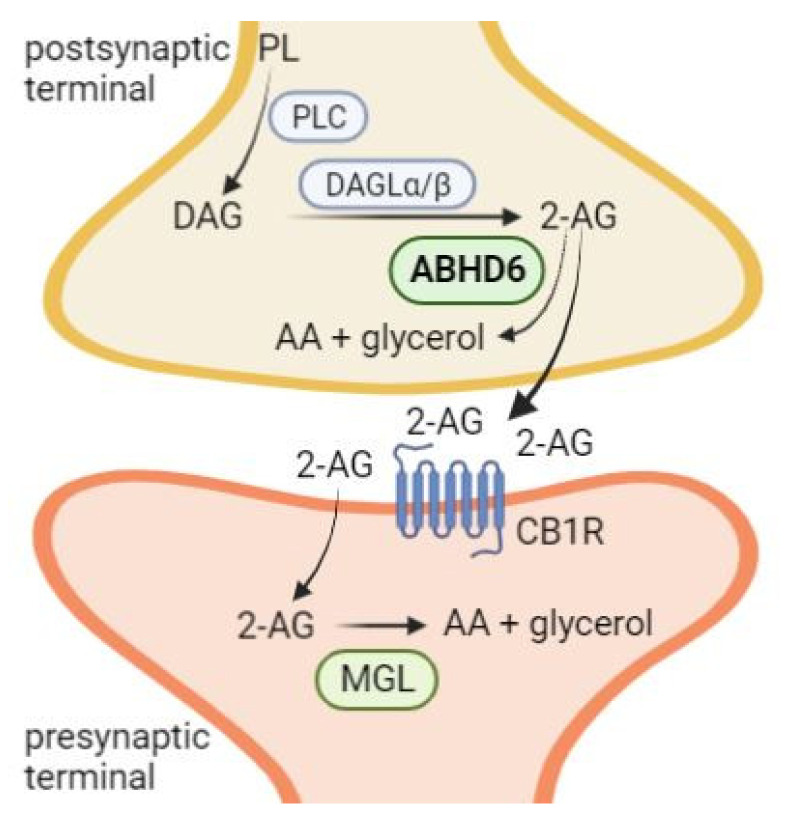
ABHD6 in endocannabinoid signaling. 2-arachidonoylglycerol (2-AG) is synthesized on membranes of postsynaptic neuron terminals by the degradation of phospholipids by phospholipase C (PLC) generating diacylglycerol (DAG). These are hydrolyzed by diacylglycerol lipase (DAGL) α and DAGLβ to form 2-AG. 2-AG is then either hydrolyzed by α/β-hydrolase domain-containing 6 (ABHD6) to arachidonic acid (AA) and glycerol or released from the cell. On postsynaptic neurons, 2-AG activates cannabinoid receptor 1 (CB1R) and is subsequently hydrolyzed by monoglyceride lipase (MGL) to AA and glycerol, terminating endocannabinoid signaling. Figure was generated using BioRender.

ABHD6 hydrolyzes the MAG 2-arachidonoylglycerol (2-AG) belonging to a class of signaling lipids, termed ECs [6]. 2-AG is synthesized from membrane phospholipids by the action of phospholipase C and two diacylglycerol lipases, DAGLα and DAGLβ. ECs are endogenous ligands of the so-called cannabinoid receptors and their effects are mimicked by the natural cannabinoid ∆^9^-tetrahydrocannabinol (∆^9^-THC), the major psychoactive component of the plant *Cannabis sativa* [32]. In contrast to other signaling lipids, ECs are produced “on demand” in response to depolarization and Ca^2+^ influx, and act in close proximity to their site of synthesis. 2-AG is a full agonist of the two major cannabinoid receptors CB1 and CB2, which are G_i/o_-protein-coupled receptors. CB1 receptor is expressed throughout the central nervous system as well as in peripheral neurons. Additionally, low expression has also been observed in non-neuronal cells. In contrast to CB1, CB2 receptor is predominantly expressed on hematopoietic cells and important for the regulation of immune response [33]. Activation of CB receptors initiates a signaling cascade that includes the activation of K^+^ channels and mitogen-activated protein kinase (MAPK), as well as the inhibition of voltage-gated Ca^2+^ channels and of adenylate cyclase, leading to lower cAMP levels. After receptor activation, ECs are taken up by cells and degraded by lipid hydrolases. Studies from the early 2000s demonstrated that MGL is the main enzyme hydrolyzing 2-AG in the brain, thereby terminating EC signaling [34]. However, in 2007, Blankman et al. suggested that ABHD6 contributes to 2-AG hydrolysis in the brain [6]. MGL-independent 2-AG hydrolysis was observed in the microglial cell line BV2, attributed to ABHD6 in subsequent studies [35,36]. Supporting evidence for a role of ABHD6 in 2-AG degradation was provided by the finding that the dual pharmacological inhibition of ABHD6 and fatty acid amide hydrolase increased 2-AG concentrations in neurons, even in the presence of MGL activity [37]. ABHD6 expression was detected in several cell types, including neurons and microglia. ABHD6 locates postsynaptically at the site of 2-AG synthesis. Therefore, it can be assumed that ABHD6 counteracts 2-AG production at postsynaptic terminals. In contrast, MGL co-localizes with CB1 receptor presynaptically and terminates 2-AG signaling (Figure 5) [35]. Thus, ABHD6 and MGL control EC signaling by degrading different 2-AG pools.

Of note, ASO-mediated knockdown or genetic deletion of ABHD6 in mice did not result in increased 2-AG levels in any tissue investigated [8,27]. However, it cannot be excluded that ABHD6 deficiency causes locally increased 2-AG levels that are not detected on an organ level. Chronic inhibition or genetic deletion of MGL affects EC signaling, which is accompanied by severe desensitization of CB1 receptors [38,39,40,41]. Conversely, ABHD6-knockout (ko) mice have not been reported to exhibit a behavioral phenotype or altered CB receptor signaling [25,27]. Together, studies suggested a rather minor contribution of ABHD6 to the regulation of 2-AG levels in vivo, possibly restricted to cells lacking MGL expression.

## 6. The Role of ABHD6 in Inflammation and Neurological Diseases

ABHD6 has been suggested to affect neurotransmission by EC-dependent and -independent mechanisms. The hydrolase has been implicated in a variety of neurological disorders, which are related to neuroinflammation. In fact, ABHD6 has been shown to control 2-AG levels in macrophages and to attenuate their activation by reducing lipopolysaccharide (LPS)-induced prostaglandin production in vitro and in vivo [42]. The underlying mechanisms are complex and not completely understood. First, ABHD6 might control 2-AG levels to activate CB2 receptors on immune cells, playing a central role in inflammatory processes [43,44]. Second, ABHD6 could control the availability of AA, the main precursor of pro-inflammatory prostaglandins [45] (see also Figure 5). Third, ABHD6 inhibition was shown to enhance the levels of the anti-inflammatory prostaglandin-D_2_-glycerol ester, which is generated by oxygenation of 2-AG by cyclooxygenase 2 (COX-2) [42]. Furthermore, ABHD6 might be implicated in systemic and tissue-specific inflammation by the regulation of (lyso)-phospholipid levels. Interestingly, lyso-phosphatidylinositol (LPI) levels are significantly increased upon systemic LPS-induced inflammation, as well as in dextran sodium sulfate- and trinitrobenzenesulfonic acid-induced colitis. Masquelier et al. showed that inhibition of ABHD6 in LPS-activated macrophages caused the accumulation of C20:4 LPI [46], which in turn can activate the non-classical CB receptor G-protein coupled receptor 55, and thereby modulate the inflammatory response [46,47,48]. Inhibition of ABHD6 has also been shown to counteract acute lung inflammation. WWL70, an ABHD6-specific inhibitor [49], significantly attenuates LPS-induced leucocyte recruitment into the lungs. This is associated with reduced expression of proinflammatory cytokines as well as increased 2-AG and lyso-phospholipid levels in bronchoalveolar lavages and lung tissue [50]. Whether the protective effect of ABHD6-inhibition is due to 2-AG mediated CB receptor activation, altered prostaglandin production or lyso-phospholipid signaling remains to be investigated.

Pharmacological inhibition of ABHD6 using the specific inhibitor WWL123 [51] exerted antiepileptic effects in mouse models of pentylenetetrazole-induced and spontaneous seizures independent of CB1 and CB2 receptor activation. Accordingly, it is reasonable to assume that the antiepileptic effect of ABHD6 blockade is mediated by a molecular species other than 2-AG [52]. These studies identify ABHD6 as an interesting therapeutic target for the treatment of epilepsy.

Other studies investigated the role of ABHD6 in models of brain injury, multiple sclerosis (MS), and neuropathic pain. Tchantchou and colleagues showed that post-injury chronic treatment of mice with traumatic brain injury using the ABHD6-inhibitor WWL70 improved motor coordination and deficits in working memory performance. ABHD6-inhibition attenuated blood–brain barrier dysfunction and neuronal degeneration by CB1 and CB2 receptor-dependent mechanisms, and reduced the generation of pro-inflammatory mediators [53]. Inhibition of ABHD6 was also protective in a mouse model of experimental autoimmune encephalomyelitis (EAE), a model for MS. MS is a chronic inflammatory disease characterized by the appearance of focal lesions with demyelination, axon degeneration, and inflammation. Treatment of mice with the ABHD6-inhibitor WWL70 elevated 2-AG levels in the cerebral cortex, downregulated the expression of pro-inflammatory markers, and attenuated macrophage and T cell infiltration in the spinal cord of EAE mice. These protective effects were at least partially mediated by the activation of CB2 receptors [44]. More recently, a protective role of ABHD6 for the treatment of MS was investigated by Manterola et al. in two studies revising the role of ABHD6 in the cuprizone model of non-immune-dependent demyelination and in EAE [54,55]. In the cuprizone model of MS, inhibition of ABHD6 using the specific inhibitor KT182 reduced demyelination and inflammation only mildly, but did not protect from oligodendrocyte excitotoxicity and maturation [54]. In this model, the protective effect of ABHD6-inhibition was less pronounced compared with the effects observed after MGL-blockade [56]. In a follow-up study, KT182 administration improved the neurological signs of EAE during disease progression; however, inflammation was not attenuated. Instead, CB1 receptor desensitization upon chronic ABHD6-blockade was observed in some brain regions, suggesting that ABHD6 might play a role in fine-tuning EC signaling under inflammatory conditions [55]. Targeting ABHD6 was also considered to be an interesting therapeutic option for the treatment of inflammatory and neuropathic pain, a complex chronic neurological disorder. Several studies emphasized a role for the EC system in the alleviation of neuropathic pain [43]. The ABHD6 inhibitor WWL70 significantly attenuated thermal hyperalgesia and mechanical allodynia induced by chronic constriction injury (CCI) in mice, independent of CB1 and CB2 receptor activation. WWL70 also reduced the inflammatory response by reduced production of pro-inflammatory cytokines, attenuated astrocyte and microglia activation, and macrophage infiltration in the central and peripheral nervous system of CCI mice. The authors proposed that the beneficial effect of WWL70 was rather caused by a reduction of prostaglandin E_2_ (PGE_2_) production than inhibition of 2-AG hydrolysis [57].

Overall, inhibition of ABHD6 produced protective effects in different mouse models of neuroinflammation-associated diseases. However, the results depend on the use of different inhibitors implicating the contribution of off-target effects, which can partially explain controversial outcomes.

Finally, ABHD6 gene expression was associated with an increased risk of systemic lupus erythematosus (SLE) in Europeans [58]. Subsequent studies revealed that ABHD6 is highly upregulated in peripheral blood mononuclear cells of SLE patients. SLE is a heterogenic autoimmune disease characterized, among others, by the presence of anti-nuclear antibodies, inflammation, vasculitis, immune complex deposition, and vasculopathy. The systemic induction of type I interferons (IFNs) plays a crucial role in SLE, and inhibition of IFN pathways is a common treatment strategy. Functional studies revealed that WWL70 significantly impaired IFNα induction by 2-AG-mediated activation of CB2 receptors [59]. These studies suggest that targeting ABHD6 in SLE might be a promising therapeutic strategy. However, it has to be considered that WWL70 has anti-inflammatory properties owing to considerable off-target inhibition of COX-2 and microsomal PGE_2_-synthase (PGES-1/2) mediating PGE_2_ biosynthesis [60]. Functional studies using ABHD6-ko mice, which would support the concept of ABHD6 inhibition as a treatment strategy for SLE, have thus far not been performed.

## 7. ABHD6 Controls Surface Delivery of AMPA-Type Glutamate Receptors

α-amino-3-hydroxy-5-methyl-4-isoxazolepropionic acid (AMPA)-type glutamate receptors are major post-synaptic receptors at excitatory synapses that mediate neurotransmission and synaptic plasticity. Mammalian cells express four types of AMPA receptor subunits (GluA1-4) and mature receptors form tetramers composed of different subunits. In addition to these central pore-forming subunits, predominantly permeable for sodium and potassium ions, AMPA receptors are associated with a variety of auxiliary proteins. AMPA receptors are responsible for the fast, immediate postsynaptic response to glutamate, whereby the composition of the pore-forming subunits and auxiliary proteins affect receptor function. In 2012, Schwenk and colleagues identified ABHD6 as component of this multiprotein complex [61]. Subsequent studies revealed that ABHD6 negatively regulates the surface delivery and affects the synaptic function of these receptors. Notably, studies using loss-of-function mutants suggested that this effect was independent of the enzymatic activity of ABHD6. Pull-down experiments confirmed that ABHD6 binds to GluA1-3, and deletion of the C-terminal domain of GluAs abolishes the interaction [62]. Overall, these observations unveiled an unexpected role of ABHD6 in receptor trafficking, which is independent of its lipid hydrolase activity. Up to now, however, these studies have not been extended to ABHD6-ko animal models, which could provide important insights into the physiological role of the enzyme in AMPA receptor-dependent synaptic transmission.

## 8. ABHD6 Affects Insulin Secretion

Insulin secretion from pancreatic β cells occurs upon fusion of insulin granules with the plasma membrane. This process is regulated by glucose and other metabolites including free fatty acids and acylglycerols. Zhao et al. investigated the role of MAGs in glucose-stimulated insulin secretion (GSIS), revealing that supplementation of cells with MAGs esterified with saturated fatty acids (C16:0 and C18:0) promote insulin release. Glucose also led to an increase in saturated MAG levels in β cells, which further increased in the presence of the ABHD6 inhibitor WWL70. This inhibitor also increased GSIS, suggesting a role of ABHD6 in the regulation of cellular MAG levels and insulin secretion. The authors suggested that MAGs bind and activate Munc13-1, a key exocytotic effector that orchestrates membrane fusion events. This activation process promotes the fusion of secretory granules with the plasma membrane, and thereby facilitates insulin secretion. GSIS was also elevated in vivo and ex vivo in mice globally lacking ABHD6 [24]. Using β-cell-specific ABHD6-ko mice, Zhao et al. demonstrated that ABHD6 is not only implicated in GSIS, but also in the control of insulin secretion promoted by various fuel stimuli (e.g., amino acids or 2-ketoisocaproate) and non-fuel stimuli such as hormones (e.g., glucagon like peptide 1 and acetylcholine) [63].

ABHD6 is highly expressed in β cells and seems to represent a major MAG hydrolase in these cells. It accounts for approximately 40% of total MAG hydrolase activity, and inhibition of ABHD6 leads to significantly increased MAG levels. However, the effect of ABHD6 inhibition on MAG accumulation is rather moderate and limited to saturated fatty acid-containing sn-1(3)-MAGs, clearly indicating that other hydrolases also contribute to MAG catabolism. Notably, Berdan et al. reported that inactivation of MGL, the major MAG hydrolase in many tissues, also affects insulin secretion in a rat insulinoma cell line (INS-1) and in rat islets. In contrast to ABHD6 inhibition, MGL blockade significantly inhibited GSIS and depolarization-induced insulin secretion. MGL-inhibition was associated with increased MAG and diacylglycerol levels, while long-chain Acyl-CoA levels were reduced in INS-1 cells [64]. These observations indicate that ABHD6 and MGL degrade different MAG pools, which contrarily affect GSIS.

## 9. The Role of ABHD6 in BMP Metabolism

BMP is a major constituent of intraluminal vesicles (ILVs) of late endosomes/lysosomes and is also present in the circulation at low concentrations [65]. It plays a key role in lipid sorting of late endosomes/lysosomes, acting as a co-factor for many lysosomal hydrolases and lipid transport molecules. Thereby, BMP can promote lysosomal lipid degradation and cholesterol export [66]. BMP is specifically important for sphingomyelin catabolism. High sphingomyelin levels can induce lysosomal damage and membrane permeabilization, ultimately leading to cell death. By stimulating acid sphingomyelinase activity, BMP can increase lysosomal stability and prevent cell death [67]. Based on these functions, it is reasonable to assume that increased BMP levels counteract the pathological accumulation of other lipids in lysosomes and lysosomal membrane permeabilization. Under pathological conditions, BMP accumulates in many genetic and drug-induced lysosomal storage disorders, as well as in the steatotic liver of mice fed a high-fat diet (HFD) [27,68]. Recent data also suggest that BMP is involved in the pathogenesis of antiphospholipid syndrome (APS), an autoimmune disease associated with arterial and venous thrombosis and pregnancy-related complications. APS is characterized by high levels of antiphospholipid antibodies, which activate coagulation pathways and induce pro-inflammatory pathways. Müller-Calleja et al. identified BMP presented by endothelial protein C receptor as a pathogenic cell surface antigen, which interacts with antiphospholipid antibodies. The authors proposed that this interaction is a central mechanism in the development and progression of autoimmune disease in patients with APS [69].

Although BMP is highly relevant for metabolic homeostasis and human disease, very little is known about the molecular basis of BMP metabolism. This lipid is resistant to lysosomal hydrolases, but efficiently degraded in the neutral pH range. ABHD6 is capable of hydrolyzing BMP at neutral pH with considerable high specific activity [13]. Furthermore, studies in mice demonstrate that ABHD6 is responsible for most of the BMP hydrolase activity detected in liver lysates, and a lack of ABHD6 increases circulating BMP levels [27]. Overall, these observations suggest a complex role of ABHD6 in BMP catabolism. ABHD6 co-localizes with late endosomes/lysosomes and the ER, suggesting that it is involved in membrane remodeling of these organelles. Yet, it is located at the cytosolic side of membranes, while BMP is enriched in the lumen of acidic vesicles. Therefore, BMP has to be exported from vesicles before being degraded by ABHD6. ILVs are very dynamic vesicles, which are produced from invaginations of the limiting membrane of endosomes. During organelle maturation, ILVs undergo substantial changes in composition and can also back-fuse with the limiting membrane. This back-fusion process represents an export route for endosomal cargo. BMP is then present at the limiting membrane and, likely together with other lipids, accessible for ABHD6. Mice lacking ABHD6 show increased circulating BMP levels, indicating that non-hydrolyzed BMP is released into the circulation, which can occur either via HDL/ApoA1-dependent mechanisms or exosomes. However, ABHD6 deficiency does not cause hepatic BMP accumulation, implicating that other intracellular enzymes are also capable of catalyzing BMP hydrolysis [27].

## 10. The Role of ABHD6 in Metabolic Syndrome

Obesity is associated with several pathological conditions, increasing the risk of developing type 2 diabetes and cardiovascular disease. In 2013, Thomas et al. showed that ABHD6 gene expression was upregulated upon HFD feeding in the liver and intestine. Antisense oligonucleotide (ASO)-mediated knock down of ABHD6 in murine liver and white adipose tissue of mice protected them from HFD-induced obesity, hepatic steatosis, and insulin resistance [8], suggesting a role of ABHD6 in the pathogenesis of metabolic syndrome. In line with this, pharmacological inhibition and global genetic deletion of ABHD6 in mice resulted in reduced body weight gain as well as improved insulin sensitivity and glucose tolerance when fed an HFD [8,25]. Mechanistically, Zhao et al. proposed that ABHD6 regulates energy homeostasis by increasing energy expenditure through adipose tissue browning and upregulation of uncoupling protein 1 in brown and white adipose tissue. Peroxisomal proliferator-activated receptor (PPAR)-α antagonists slightly reversed the observed phenotype [25]. The authors suggested that this phenotype is caused by the accumulation of sn-1-MAG in ABHD6-deficient adipose tissue activating PPARα and γ. Further evidence for a role of ABHD6 in the regulation of energy homeostasis was provided by a study using adipose tissue-specific ABHD6-ko mice. These mice show increased whole-body insulin sensitivity, while glucose homeostasis and body composition remained unchanged. After cold exposure, adipose tissue-specific ABHD6-ko mice show increased energy expenditure and resistance to hypothermia [28].

The EC system plays an important role in the control of feeding and energy expenditure in the ventromedial hypothalamus (VMH) [70]. Based on this observation and the ability of ABHD6 to degrade 2-AG, Fisette et al. investigated the role of the enzyme in VMH neurons in the control of energy balance. Deletion of ABHD6 in this brain area increased VMH 2-AG levels in fasted mice, but not in fed mice, and this was associated with metabolic deficits. VMH-specific ABHD6-ko mice showed blunted basal and fasting-induced food intake. Notably, some effects were contrary to the phenotype observed in global or adipose-specific ABHD6-ko mice. Mice exhibited reduced energy expenditure and cold-induced thermogenesis, and were prone to HFD-induced obesity. The authors suggested that the EC system in VMH neurons regulates the adaption to metabolic challenges and the lack of ABHD6 in these neurons blunts the metabolic flexibility of mice [26]. These observations could be important for the development of ABHD6 inhibitors and for the interpretation of their therapeutic effects in metabolic disorders. Apparently, inhibition of ABHD6 in peripheral tissues counteracts obesity and co-morbidities, while ABHD6 inactivation in VMH neurons induces opposite effects.

## 11. ABHD6 and Cancer

Even before its biochemical characterization, ABHD6 was found to be differentially expressed in tumor cell lines, ranging from very high expression in U2OS (bone), PC-3 (prostate), and Jurkat (leukocyte) cells, to absent expression in Hela (cervical) and U251 (brain) cells [71]. Subsequent studies suggested that ABHD6 is highly abundant in Ewing family tumor cell lines and might be an interesting new diagnostic or therapeutic target. However, knock down of ABHD6 did not affect the growth of Ewing tumor cells in vitro [72].

Grüner et al. used the murine pancreatic ductal adenocarcinoma (PDAC) cell line (0688M) to study the effect of hydrolase inhibitors on pancreatic cancer metastatic seeding in vivo. By screening ~700 hydrolase inhibitors, they identified several hits reducing the metastatic fitness of PDAC cells [73]. Interestingly, among the top hits was a compound targeting ABHD6. Subsequent investigations using more selective inhibitors and genetic approaches confirmed that ABHD6 promotes metastatic seeding. Evidence for a pro-oncogenic function of ABHD6 also came from a study investigating the role of ABHD6 in the pathogenesis of non-small cell lung carcinoma (NSCLC) [74]. ABHD6 silencing and pharmacological inhibition reduced migration and invasion of NSCLC cells in vitro. Furthermore, ABHD6 inhibition blunted metastatic seeding and tumor growth in mice. The authors observed substantial upregulation of ABHD6 in tumor tissues and proposed that lack of ABHD6 leads to the accumulation of MAG, promoting cancer aggressiveness. Overall, these studies identify ABHD6 as an interesting target for the development of anti-metastatic and anti-tumor therapies.

## 12. Conclusions

**Figure 6:**
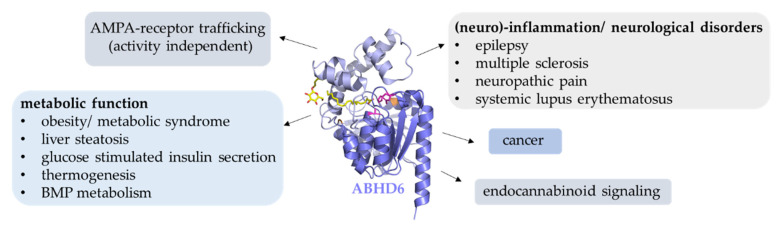
ABHD6 is involved in multiple (patho)-physiological processes. ABHD6 has been shown to be implicated in various (patho)-physiological processes, including lipid degradation, lipid signaling, inflammation, obesity, and cancer.

ABHD6 is a ubiquitously expressed enzyme, and its biochemical and physiological functions are only now being characterized in detail. It hydrolyzes a variety of lipid substrates such as MAGs, lyso-phospholipids, and BMP, and has been implicated in different physiological processes including insulin secretion, receptor trafficking, membrane remodeling, and synaptic transmission (Figure 6). It must also be considered that ABHD6 is a membrane-associated protein and likely plays a role in lipid remodeling of membranes. Structural data indicate that ABHD6 preferentially localizes at negatively charged membrane sites. The phospholipid composition and the charge of membranes can substantially affect the interaction of membranes with proteins. Negatively charged lipids play a critical role in regulating vesicular transport and endosome maturation. Thus, it remains to be investigated whether changes in membrane composition and charge also contribute to the multiple effects on metabolism, trafficking, and inflammation.

Pharmacological inhibition of ABHD6 causes beneficial metabolic effects in mice such as enhancement of insulin secretion and energy expenditure, thus qualifying ABHD6 as a promising target for the treatment of the metabolic syndrome and its comorbidities. Furthermore, blockade of ABHD6 has neuro-protective, anti-inflammatory, and anti-oncogenic effects, identifying the enzyme as also being an interesting pharmacological target for the treatment of (neuro)-inflammatory and neurodegenerative disorders. Current evidence suggests that these effects are mediated by lipid signaling molecules accumulating in the absence of ABHD6 activity. However, some of these observations possibly derive from off-target effects of ABHD6 inhibitors and have to be confirmed in genetic ABHD6-ko models. Moreover, detailed structural analysis of ABHD6 will support the development of highly specific inhibitors. In summary, the therapeutic potential of ABHD6 inhibition for various disease treatments is vast and promising, but warrants further research.

## Figures and Tables

**Table 1:** Mouse and rat lines with global or tissue-specific deletion of ABHD6.

Organism/Strain	Knockout/Knockdown	Observation	Reference
ABHD6-ko (mouse)	global	increased glucose stimulated insulin secretion;	[24]
ABHD6-ko (mouse)	global	increased circulating bis(monoacylglycerol)phosphate levels;	[27]
ABHD6-flox/Ins1-Cre/ERT(mouse)	β-cell-specific	enhanced glucose stimulated insulin secretion in vivo and ex vivo;	[24]
ABHD6-flox/AdipoQ-Cre/ERT2(mouse)	adipocyte-specific	elevated energy expenditure in cold and resistance to cold-induced hypothermia; protected from diet-induced obesity;	[28]
ABHD6-ko (rat)	global (CRISPR/Cas9-mediated)	shorter intervals between bladder contractions, hyperalgesia and increased PGE_2_;	[29]
ABHD6-ASO (mouse) ^1^	liver-specific (ASO-mediated knock down)	protected from diet induced obesity and liver steatosis;	[8]
VMH^KO^ (mouse) ^2^	VMH neuron-specific (AAV-mediated)	altered food intake, reduced energy expenditure, prone to diet-induced obesity;	[26]

^1^ ASO, antisense oligonucleotide; ^2^ VMH, ventromedial hypothalamus.

## Data Availability

Not applicable.
